# Genetic Structure of Human A/H1N1 and A/H3N2 Influenza Virus on Corsica Island: Phylogenetic Analysis and Vaccine Strain Match, 2006–2010

**DOI:** 10.1371/journal.pone.0024471

**Published:** 2011-09-14

**Authors:** Alessandra Falchi, Jean Pierre Amoros, Christophe Arena, Jean Arrighi, François Casabianca, Laurent Andreoletti, Clément Turbelin, Antoine Flahault, Thierry Blanchon, Thomas Hanslik, Laurent Varesi

**Affiliations:** 1 INSERM, UMR-S 707, F-75012, Paris, France; 2 University Pierre and Marie Curie, Université Paris 06, UMR-S U707, F-75012, Paris, France; 3 Laboratory of Virology, University of Corsica, Corsica, France; 4 Observatoire Régionale de la Santé, Ajaccio, France; 5 Institut national de la Recherche Agronomique, Corte, France; 6 Unité de Virologie Médicale et Moléculaire, Centre Hospitalier Universitaire, Reims, France; 7 IFR 53/EA-4303 (DAT/PPCIDH), Faculté de Médecine, Reims, France; 8 EHESP School of Public Health, Rennes, France; University of Liverpool, United Kingdom

## Abstract

**Background:**

The aim of this study was to analyse the genetic patterns of Hemagglutinin (HA) genes of influenza A strains circulating on Corsica Island during the 2006–2009 epidemic seasons and the 2009–2010 pandemic season.

**Methods:**

Nasopharyngeal samples from 371 patients with influenza-like illness (ILI) were collected by General Practitioners (GPs) of the *Sentinelles* Network through a randomised selection routine.

**Results:**

Phylogenetic analysis of HA revealed that A/H3N2 strains circulating on Corsica were closely related to the WHO recommended vaccine strains in each analyzed season (2006–2007 to 2008–2009). Seasonal Corsican influenza A/H1N1 isolated during the 2007–2008 season had drifted towards the A/Brisbane/59/2007 lineage, the A/H1N1 vaccine strain for the 2008–2009 season. The A/H1N1 2009 (A/H1N1pdm) strains isolated on Corsica Island were characterized by the S220T mutation specific to clade 7 isolates. It should be noted that Corsican isolates formed a separate sub-clade of clade 7 as a consequence of the presence of the fixed substitution D222E.

The percentages of the perfect match vaccine efficacy, estimated by using the *p*_epitope_ model, against influenza viruses circulating on Corsica Island varied substantially across the four seasons analyzed, and tend to be highest for A/H1N1 compared with A/H3N2 vaccines, suggesting that cross-immunity seems to be stronger for the H1 HA gene.

**Conclusion:**

The molecular analysis of the HA gene of influenza viruses that circulated on Corsica Island between 2006–2010 showed for each season the presence of a dominant lineage characterized by at least one fixed mutation. The A/H3N2 and A/H1N1pdm isolates were characterized by multiples fixation at antigenic sites. The fixation of specific mutations at each outbreak could be explained by the combination of a neutral phenomenon and a founder effect, favoring the presence of a dominant lineage in a closed environment such as Corsica Island.

## Introduction

Type A influenza viruses are major pathogens for humans. Influenza A virus is further classified based on the antigenic properties of its surface glycoproteins, the Haemagglutinin (HA) and the Neuraminidase (NA) [1]. Influenza A/H3N2 and A/H1N1 subtypes are the major subtypes currently circulating in human populations [2]. HA is of special interest due to its role in the viral entry mechanism and immune recognition. It consists of two subunits: HA1, which contains the receptor-binding and antigenic domains, and the HA2 subunit, which is responsible for the fusion of the virion with the endosomal membrane of the host cell [3]. The HA1 subunit undergoes a process termed positive Darwinian selection through continuous antigenic mutations that allow the virus to evade the host's humoral immune response [4].

This process is called antigenic drift. Variants that best escape the host immune response are thought to have a significant reproductive advantage [5]. Specifically, the variable antigenic regions of the HA1 domain are potential targets of neutralising antibodies, and thus amino acid substitutions at these regions (A to E) have been associated with annual epidemics in humans. In contrast, the amino acids within the receptor binding site (RBD) of the HA1 domain are relatively conserved. Variations have also been observed in the N-linked glycosylation sites of HA, with some strains having lost or gained a glycosylation site and potentially an altered glycoprotein function. Concerning the NA, antigenic variations usually occur in the catalytic or framework sites of the protein.

Another process, called reassortment, is also considered to be a major force in the evolution of influenza virus [5]. It occurs when the virus acquires an HA of a different influenza virus subtype via reassortment of one or more gene segments, and this is thought to be the basis for the more devastating influenza pandemics [6]. New influenza pandemics may emerge through reassortment with strains from swine or avian reservoirs [7].

The first influenza pandemic of this century was declared in April of 2009, with the emergence of a novel H1N1 influenza A virus strain in Mexico and the USA [8], [9]. In France, the A/(H1N1)2009 pandemic wave spanned from September 6, 2009 to December 26, 2009 (www.sentiweb.fr ). During the epidemic, the pandemic strain was almost exclusively found among influenza virus isolates [10].

The effectiveness of annual influenza vaccines depends on the selection of component strains that offer optimal immunity from the numerous variants in the global influenza virus circulation. Studies based on sequencing analyses of viruses can be utilised as surveillance tools and can contribute to the vaccine selection process when they are combined with classical serological antigenic analysis.

The aim of this study was to analyse the genetic patterns of HA genes of influenza A strains circulating on Corsica Island during the 2006–2009 epidemic seasons and the 2009–2010 pandemic season.

## Methods

### Ethics Statement

Based on French national laws ethical permissions (Law 1121- 1- 1° R. 1121- 2) are not required for specific microbiological diagnostics treatment of the patients and further characterization of the viruses. All samples were coded and tested anonymously. Patient information was stored according to national regulations and access to such data was restricted (permissions CNIL 471393).

### Clinical samples and epidemic data

**Figure 1:**
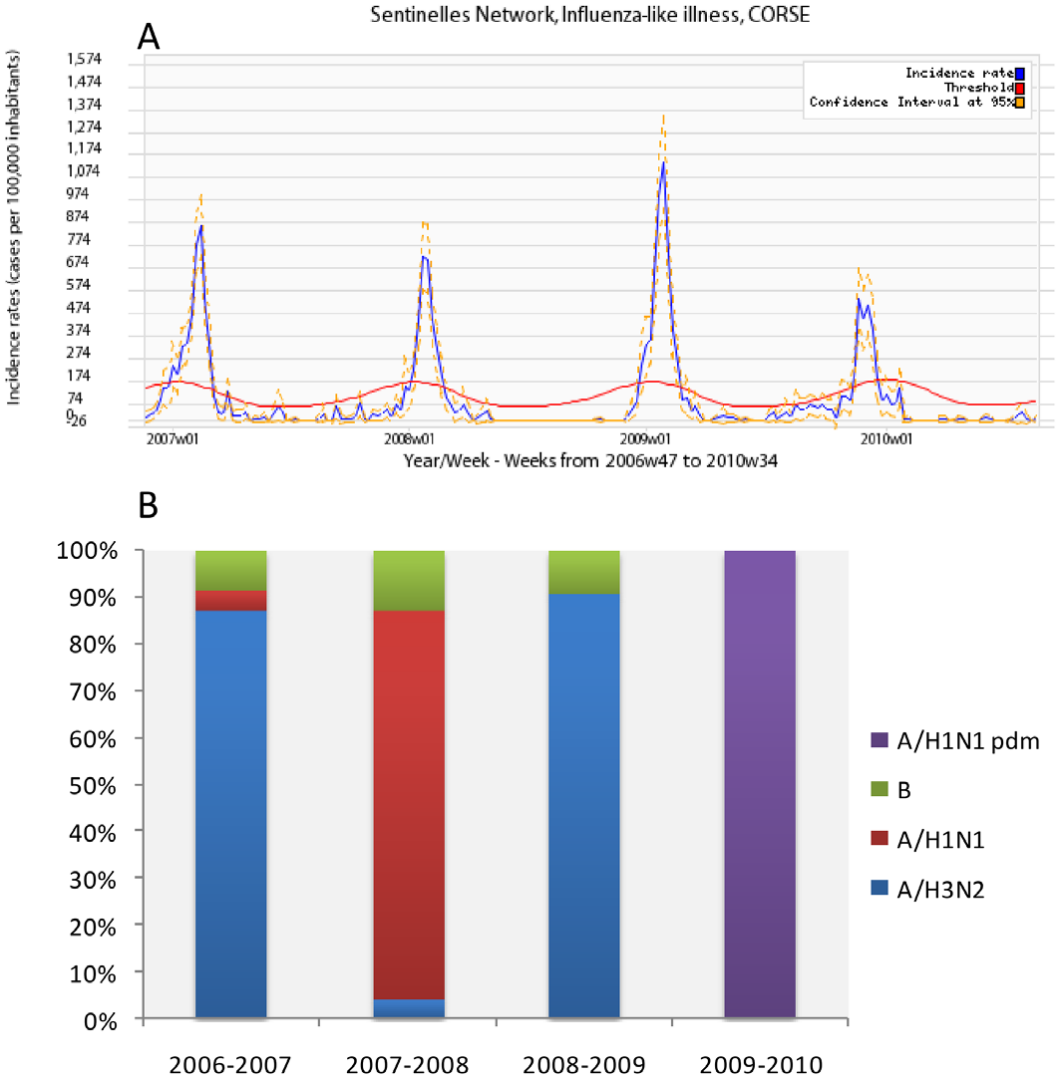
Weekly influenza epidemic time series. (A). Influenza seasons in Corsica Island. Incidence rate (cases per 100,000 inhabitants) in blue. In red, the epidemic threshold since 2006–2007 season calculated by a periodic regression model applied to the former observed data (Serfling's method). In yellow confidence interval at 95%. (B) Proportions of influenza viruses A/H1N1 pdm; A/H1N1; A/H3N2 and B in Corsica islands.

Three hundred and seventy one samples from patients with influenza-like illness (ILI) were collected by *Sentinelles* General Practitioners (GPs) of the *Sentinelles* Network starting with the 2006–2007 season (Fig. 1). All cases represented mild infections. Nasopharyngeal swabs and clinical data were collected through a randomised selection routine. Doctors included the first patient of each week, of any age. Corsican *Sentinelles* GPs swabbed patients presenting an ILI according to the *Sentinelles* definition: “sudden onset of fever >39°C (>102°F) with respiratory signs and myalgia” [11].

The nasopharyngeal swabs were sent by mail in 2 ml of viral transport medium within two days to the virological laboratory. Detailed demographic and clinical data (the time of onset of symptoms, reported symptoms, physical findings and influenza vaccination status) were obtained from patients during the medical visit.

### RT-PCR Assays for Influenza Virus Detection and Subtyping

After processing the clinical materials by the standard laboratory procedures, the individual sample was inoculated in the Madin Canine Kidney (MDCK) cell lines for the isolation of viruses. The inoculated MDCK cell line was observed at 34°C for cytopathic effects. The viruses were passaged three times to obtain sufficient virus titers for virus identification.

RNA was extracted from 140 µl of clinical sample and of the supernatant using the QIAmp Viral RNA Mini kit® (QIAGEN, Courtaboeuf, France). Multiplex RT-PCR assays were performed using the One-step RT-PCR kit® (QIAGEN, Courtaboeuf, France) and using primers based on the M, HA and NA glycoprotein gene sequences of seasonal influenza A/H1N1 and A/H3N2 viruses [12]–[14]. Positive and negative controls were included in each multiplex RT-PCR.

In order to detect and assign the A/H1N1pdm strains isolated from patients, a real-time PCR assay was performed using a specified RT-PCR protocol provided by the French National Influenza Centre [15]. Positive and negative controls were included in each RT-PCR run.

For the detection of A/H1N1pdm, the M RT-PCR was associated with a specific RT-PCR targeting the HA gene. This strategy is pertinent in the context of a pandemic as the new virus may drift, mainly due to mutation in hemagglutinin and/or neuraminidase genes, but variants will still be detected by this universal M RT-PCR [15].

### Nucleotide sequencing

The HA sequences were amplified using the primer set for A/H1N1, A/H1N1pdm and A/H3N2 influenza virus (primers sequences are available on request). The resulting amplicons were analyzed by 2% agarose gel electrophoresis. Double-stranded sequencing of the purified PCR products was performed using an Applied Biosystems Sequencer (ABI 3700 Perkin- Elmer).

### Nucleotide sequence accession numbers

The nucleotide sequence data from this study were deposited with the following accession numbers: for A/H1N1 isolates between JF701798 and JF701831; for A/H3N2 isolates between JF701832 and JF711877 and for A/H1N1pdm isolates between JF01878 and JF701906.

### Phylogenetic analysis

Nucleotide sequences were aligned with the CLUSTAL X [16] program for the major coding regions of the three segments: HA (875 bp) for A/H3N2 isolates from 2007–2008 and 2008–2009, HA (1056 bp) for A/H1N1 isolates from 2006–2007 and 2007–2008 and HA (1018 bp) for A/H1N1pdm from 2009–2010. The Find Model program [17] was used to identify the optimal evolutionary model that best fitted our sequence dataset. Akaike Information Criteria and the hierarchical likelihood ratio test indicated that the HKY+Γ model was the best fit to the data. To infer the evolutionary relationship for the influenza virus analyzed, we employed the Maximum likelihood (ML) trees constructed under the HKY+Γ model using software from the PhyML program [18].

The genomic sequences of the vaccine strains and the other strains used in this study were obtained from the Influenza Sequences Database (http://www.flu.lanl.gov).

### Nucleotide diversity

The average pairwise nucleotide diversity among sequences was estimated for the A/H3N2, the A/H1N1 seasonal influenza viruses and for the A/H1N1 pdm virus population. For this analysis, a maximum likelihood (ML) method was employed, available in the MEGA4 package [19], with the standard deviation estimated using 100 bootstrap replicates.

### Prediction of N-Glycosylation

Potential N-glycosylation sites (amino acids Asn-X-Ser/Thr, where X is not Asp or Pro) were predicted using nine artificial neural networks with the NetNGlyc server 1.0 [20]. A threshold value of 0.5 average potential score was set to predict glycosylated sites.

### Estimation of vaccine efficacy (VE) using the p_epitope_ model

Antigenic distance is a quantity that should define difference of viral strains, as determined by the human immune system. The *p*_epitope_ sequence-based method has been shown to be an effective antigenic distance measure between two influenza viral strains [21]–[24]. The meta-analysis of epidemiological human VE studies shows that the single dominant epitope is the critical region that determines the epidemiological VE [21]–[24]. There are five non-overlapping epitopes on the surface of H3 and H1 HA molecule, namely epitopes A–E, to which different sets of antibodies bind. In each epitope, the p value is defined as the fraction of mutated amino acids [21]. The dominant epitope is defined as the epitope with the greatest p value. The greatest p value is *p*_epitope_ [21].

Epidemiological data on the vaccine efficacies in previous flu seasons when A/H3N2 or A/H1N1 subtype was dominant were collected [21]–[24]. The identities of the vaccine strains and dominant circulating strains were also obtained to calculate *p*_epitope_.

The A/H3N2 and A/H1N1 VE correlates with *p*_epitope_ with R2 = 0.81 and R2 = 0.68, respectively. This correlation shows that *p*_epitope_ defined by the single dominant epitope is a quantitative definition of antigenic distance.

The relation between VE and *p*_epitope_ is given by E = −2.47×*p*_epitope_+0.47 (1) for A/H3N2 influenza virus and by E = −1.19×*p*_epitope_+0.53 (2) for A/H1N1 strains. These two fitted models predict a vaccine effectiveness of 47% when *p*_epitope_ = 0 for A/H3N2 and of 53% when *p*_epitope_ = 0 for A/H1N1 [21], [23].

The comparison between A/H3N2 and A/H1N1 vaccine effectiveness illustrates that A/H1N1 vaccine has higher effectiveness than the A/H3N2 vaccine as a function of *p*_epitope_. This observation suggests that the host immune system is more effective at recognizing and eliminating the A/H1N1 virus (*p*_epitope_ = 0), and that humoral cross-immunity is stronger for H1 HA (*p*_epitope_>0).

By considering only the substitutions that occur in the epitopes, we calculated the value of the p_epitope_ (as the number of mutations within an antibody antigenic site divided by the number of amino acids defining the site), between the influenza virus strains representatives of the dominant lineage isolated in Corsica Island with respect to the vaccine strain of each corresponding season. The antigenic epitope which has the greatest percentage of mutations was considered as dominant. Percentages of the perfect match VE were estimated by using the equation 1 and 2 described precedently.

## Results

### Prevalence of influenza viruses on Corsica Island from 2006 to 2010

A total of 371 samples were collected and analyzed at the *Sentinelles* site on Corsica Island in the four-year period from 2006–2010. Influenza-like illness activity and the relative prevalence of influenza virus subtypes from season to season on Corsica Island are shown in Fig. 1 (A) and (B), respectively.

In 2006–2007 [25], 134 samples were collected from February 2^nd^ to March 16^th^ 2007. Out of 134 samples, 93 (69.4%) were positive and 85 (91.4%) were Influenza type A, whereas influenza B was detected in 8 (6%) of 93 patients. Of the 85 influenza A strains identified, 81 (95%) were sub-typed as A/H3N2 and 4 (5%) were sub-typed as A/H1N1.

In 2007–2008, 85 samples were collected from January 30^th^ to February 28^th^ 2008. Out of 85 samples, 70 (83%) were positive; 61 (87%) were influenza type A and 9 (13%) were typed as influenza B. Of the 61 influenza A strains identified, 58 (95%) were sub-typed as A/H1N1 and 3 (5%) were sub-typed as A/H3N2.

In 2008–2009, 98 samples were collected from November 2^nd^ 2008 to February 18^th^ 2009. Out of 98 samples, 64 (65%) were positive; 58 (91%) were Influenza A and 6 (9%) were influenza B. Of the 58 influenza A strains identified, 100% were sub-typed as A/H3N2.

In 2009–2010, 54 samples were collected from November 3^rd^ 2009 to January 11^th^ 2010. Out of the 54 samples, 33 (61%) were positive. All positive samples were A/H1N1pdm influenza viruses.

### Influenza A/H3N2 viruses

The HA sequences of the A/H3N2 viruses circulating on Corsica Island during the 2006–2007 (N = 16), 2007–2008 (N = 2) and the 2008–2009 seasons (N = 45) were compared with the vaccine strains.

Phylogenetic analysis showed that HA sequences of the A/H3N2 isolates formed clusters representative for the 2006–2007, 2007–2008 and 2008–2009 seasons.

**Figure 2:**
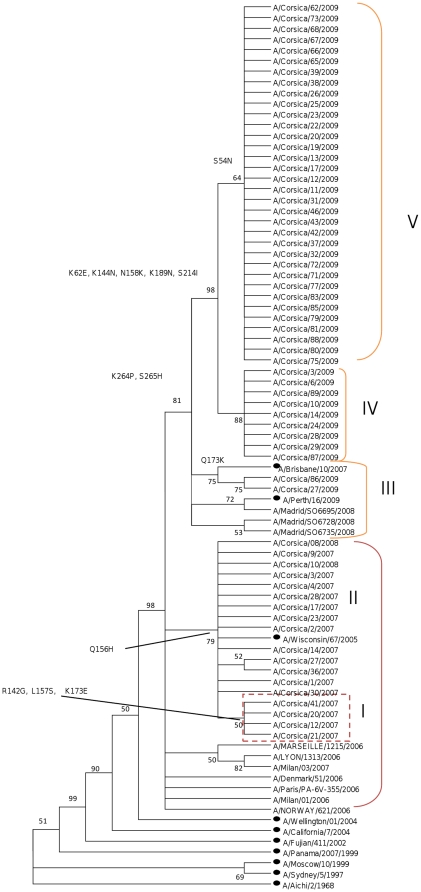
Phylogenetic tree of the HA domain of the A/H3N2 strains from Corsica Island. Maximum likelihood phylogenetic tree analysis of HA genes of A/H3N2 strains circulating in Corsica Island from 2006 to 2009 (• represents vaccine strains).

As previously reported [25], the HA sequences of the A/H3N2 isolated during the 2006–2007 season appeared to be closely related to the A/Wisconsin/67/2005-like lineage (vaccine strain for 2006–2007 and 2007–2008 in the Northern Hemisphere) (Fig. 2). All isolates were characterized by the substitution H156Q (Fig. 2) with respect to A/Wisconsin/67/2005. Several strains were characterized by H156Q associated with at least one of these three additional substitutions: R142G, L157S and K173E (clade I; Fig. 2).The nucleotide diversity of the A/H3N2 2006–2007 population was 0.002±0.001. The nucleotide identities between the 2006–2007 strains and the A/Wisconsin/67/2005-like lineage were 99.7% and 99.4% based on amino acids.

The A/H3N2 viruses isolated on Corsica Island during the 2007–2008 season clustered with the A/H3N2 viruses isolated during the 2006–2007 season and were related to A/Wisconsin/67/2005. These two strains were not characterized by the mutation H156Q (Fig. 2; Table 1). The nucleotide identities between the 2007–2008 strains and the A/Wisconsin/67/2005-like lineage were 99.6% and 99.4% based on amino acids. The nucleotide identities between the 2007–2008 strains and the A/Brisbane/10/2007-like lineage (vaccine strain for the 2008–2009 season in the Northern Hemisphere) were 99.8% and 88.6% based on amino acids.

**Table 1:** Amino acid substitution in A/H3N2 influenza viruses isolated in Corsica Island from 2008 to 2009.

Amino acid position	50	54	62	122	138	140	144	156	158	173	186	189	194	214	223	264	265
**Antigenic sites**	**C**			**A**	**A**	**A**	**A**	**B**	**B**	**D**	**B**	**B**	**D**	**D**		**E**	**E**
**A/Perth/16/2009**	**E**	**S**	**K**	**N**	**A**	**I**	**K**	**H**	**N**	**Q**	**G**	**K**	**L**	**S**	**V**	**K**	**S**
**A/Brisbane/10/2007**	**.**	**.**	**E**	**.**	**.**	**.**	**N**	**.**	**K**	**K**	**.**	**N**	**.**	**I**	**.**	**.**	**.**
**A/Wisconsin/67/2005**	**G**	**.**	**E**	**D**	**S**	**K**	**N**	**H**	**K**	**K**	**V**	**N**	**.**	**I**	**I**	**.**	**.**
A/Corsica/3/2009	.	.	E	.	.	.	N	.	K	.	.	N	.	I	.	P	H
A/Corsica/79/2009	.	.	E	.	.	.	N	.	K	.	.	N	.	I	.	P	H
A/Corsica/85/2009	.	.	E	.	.	.	N	.	K	.	.	N	.	I	.	P	H
A/Corsica/86/2009	.	.	E	.	.	.	N	.	K	K	.	N	P	I	.	.	.
A/Corsica/6/2009	.	.	E	.	.	.	N	.	K	.	.	N	.	I	.	P	H
A/Corsica/89/2009	.	.	E	.	.	.	N	.	K	.	.	N	.	I	.	P	H
A/Corsica/83/2009	.	.	E	.	.	.	N	.	K	.	.	N	.	I	.	P	H
A/Corsica/77/2009	.	.	E	.	.	.	N	.	K	.	.	N	.	I	.	P	H
A/Corsica/71/2009	.	N	E	.	.	.	N	.	K	.	.	N	.	I	.	P	H
A/Corsica/72/2009	.	.	E	.	.	.	N	.	K	.	.	N	.	I	.	P	H
A/Corsica/32/2009	.	N	E	.	.	.	N	.	K	.	.	N	.	I	.	P	H
A/Corsica/37/2009	.	.	E	.	.	.	N	.	K	.	.	N	.	I	.	P	H
A/Corsica/42/2009	.	N	E	.	.	.	N	.	K	.	.	N	.	I	.	P	H
A/Corsica/43/2009	.	N	E	.	.	.	N	.	K	.	.	N	.	I	.	P	H
A/Corsica/46/2009	.	N	E	.	.	.	N	.	K	.	.	N	.	I	.	P	H
A/Corsica/31/2009	.	N	E	.	.	.	N	.	K	.	.	N	.	I	.	P	H
A/Corsica/10/2009	.	.	E	.	.	.	N	.	K	.	.	N	.	I	.	P	H
A/Corsica/11/2009	.	.	E	.	.	.	N	.	K	.	.	N	.	I	.	P	H
A/Corsica/12/2009	.	.	E	.	.	.	N	.	K	.	.	N	.	I	.	P	H
A/Corsica/17/2009	.	N	E	.	.	.	N	.	K	.	.	N	.	I	.	P	H
A/Corsica/13/2009	.	N	E	.	.	.	N	.	K	.	.	N	.	I	.	P	H
A/Corsica/14/2009	.	.	E	.	.	.	N	.	K	.	.	N	.	I	.	P	H
A/Corsica/19/2009	.	N	E	.	.	.	N	.	K	.	.	N	.	I	.	P	H
A/Corsica/20/2009	.	N	E	.	.	.	N	.	K	.	.	N	.	I	.	P	H
A/Corsica/22/2009	.	N	E	.	.	.	N	.	K	.	.	N	.	I	.	P	H
A/Corsica/23/2009	.	N	E	.	.	.	N	.	K	.	.	N	.	I	.	P	H
A/Corsica/24/2009	.	.	E	.	.	.	N	.	K	.	.	N	.	I	.	P	H
A/Corsica/25/2009	.	.	E	.	.	.	N	.	K	.	.	N	.	I	.	P	H
A/Corsica/26/2009	.	.	E	.	.	.	N	.	K	.	.	N	.	I	.	P	H
A/Corsica/27/2009	.	.	E	.	.	.	N	.	K	K	.	N	P	I	.	.	.
A/Corsica/28/2009	.	.	E	.	.	.	N	.	K	.	.	N	.	I	.	P	H
A/Corsica/29/2009	.	.	E	.	.	.	N	.	K	.	.	N	.	I	.	P	H
A/Corsica/38/2009	.	.	E	.	.	.	N	.	K	.	.	N	.	I	.	P	H
A/Corsica/39/2009	.	.	E	.	.	.	N	.	K	.	.	N	.	I	.	P	H
A/Corsica/65/2009	.	.	E	.	.	.	N	.	K	.	.	N	.	I	.	P	H
A/Corsica/66/2009	.	.	E	.	.	.	N	.	K	.	.	N	.	I	.	P	H
A/Corsica/67/2009	.	N	E	.	.	.	N	.	K	.	.	N	.	I	.	P	H
A/Corsica/68/2009	.	N	E	.	.	.	N	.	K	.	.	N	.	I	.	P	H
A/Corsica/62/2009	.	N	E	.	.	.	N	.	K	.	.	N	.	I	.	P	H
A/Corsica/73/2009	.	N	E	.	.	.	N	.	K	.	.	N	.	I	.	P	H
A/Corsica/81/2009	.	N	E	.	.	.	N	.	K	.	.	N	.	I	.	P	H
A/Corsica/88/2009	.	N	E	.	.	.	N	.	K	.	.	N	.	I	.	P	H
A/Corsica/87/2009	.	.	E	.	.	.	N	.	K	.	.	N	.	I	.	P	H
A/Corsica/80/2009	.	.	E	.	.	.	N	.	K	.	.	N	.	I	.	P	H
A/Corsica/75/2009	.	.	E	.	.	.	N	.	K	.	.	N	.	I	.	P	H
A/Corsica/08/2008	G		E	D	S	K	N		K	K	V	N		I	I		
A/Corsica/10/2008	G		E	D	S	K	N		K	K	V	N		I	I		

The HA sequences of A/H3N2 viruses isolated on Corsica Island during the 2008–2009 season had drifted from the A/Wisconsin/67/2005-like lineage (98.1% and 97% similarity based on nucleotide and amino acids sequences, respectively). These strains were closely to the A/Brisbane/10/2007-like lineage (vaccine strain for the 2008–2009 season in the Northern Hemisphere) (Fig. 2; Table 1). The nucleotide identities between the 2009 strains and the A/Brisbane/10/2007-like lineage were 99.3% and 98.9% based on amino acids. The nucleotide identities between the 2009 strains and the A/Perth/16/2009-like lineage (vaccine strain for the 2009–2010 season in the Northern Hemisphere) were 98.2% and 96.2% based on amino acids.

The H3 nucleotide sequences of the 2009 virus isolates clustered into three sub-clades (III, IV and V). All groups were characterized by two variations (K264P and S265H) compared to the vaccine strain A/Brisbane/10/2007 (vaccine strain for the 2008–2009 season in the Northern Hemisphere) and by five altered amino acids (K62E, K144N, N158K, K189N and S214I) with respect to A/Perth/16/2009 (vaccine strain for the 2009–2010 season in the Northern Hemisphere). The sub-clade V was characterized by the supplementary mutation S54N when compared to A/Brisbane/10/2007 and A/Perth/16/2009 (Fig. 2). The sub-clade III was characterized by the Q173K mutation with respect to A/Perth/16/2009. The sub-clade IV showed one supplementary variation compared to A/Perth/16/2009 (Q173K and L194D). The K144N mutation was located at antigenic site A, while the N158K and K189N mutations were located at site B, the S241I and Q173K mutations were at site D and the K264P and S265H mutations were at site E.

The nucleotide diversity of this A/H3N2 2008–2009 population was 0.005±0.002. The amino acids at the terminal SA of all H3 isolates were Ile (I)-226 and Ser (S)-228.

Nine potential N-glycosylation sites were predicted in HA1 of the H3 subtype (8, 22, 38, 63, 126, 133, 165, 246 and 285) with a threshold >0.5. These sites were found to be conserved in all isolates obtained during the 2006–2009 seasons. Strains isolated in 2007–2008, 2008–2009 and ten strains (4.2%; 10/42) isolated in 2006–2007 had lost glycosylation at position 144.

### Influenza A/H1N1 virus

**Figure 3:**
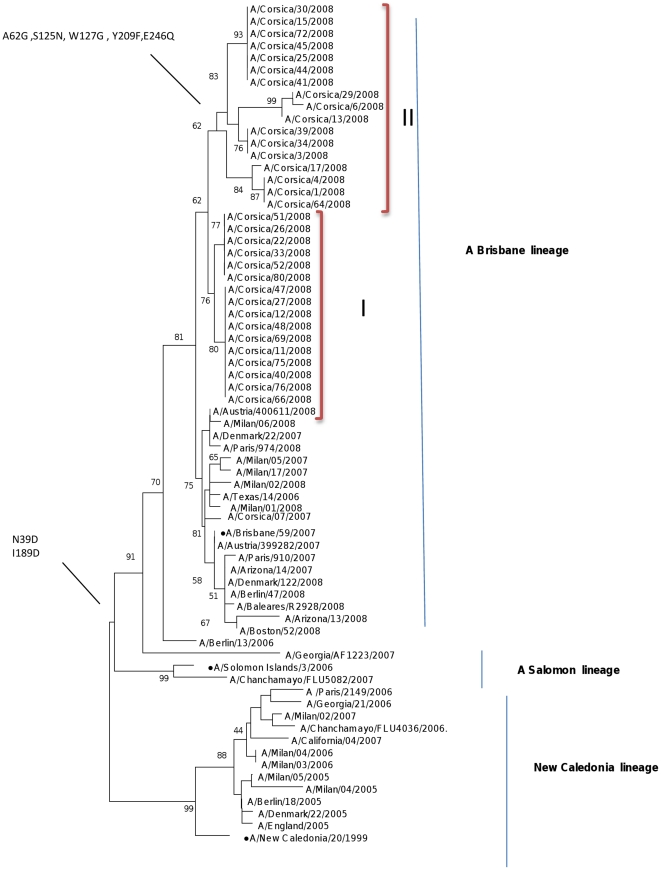
Phylogenetic tree of the HA domain of the A/H1N1 strains from Corsica Island. Maximum likelihood phylogenetic tree analysis of HA genes of A/H1N1 strains circulating in Corsica Island during 2007–2008 season (• represents vaccine strains).

A/H1N1 isolates from the 2006–2007 (N = 1) and 2007–2008 (N = 34) season were compared with vaccine strains for phylogenetic analysis (Fig. 3; Table 2). The HA sequences of influenza viruses isolated on Corsica Island during the 2007–2008 season were separated into two sub-clusters (I and II) as shown in Fig. 3. Both clades were characterized by the N39D and I189D (antigenic site B) with respect to A/Brisbane/59/2007 (vaccine strain of the 2008–2009 season in the Northern Hemisphere) (Table 2). Clade II was characterized by the A62G, S125N (antigenic site B), W127G, C139S and S140C (antigenic site A), Y209F (at antigenic site D) and E246Q (Table 2). The isolate A/Corsica/7/2007 was characterized by the substitutions I189D (antigenic site B) and G240R with respect to A/Brisbane/59/2007 (Fig. 3; Table 2).

**Table 2:** Amino acid substitution in A/H1N1 influenza viruses isolated in Corsica Island from 2007 to 2008.

Amino acid positions	39	62	125	127	139	140	189	209	240	246
**Antigenic sites**			**B**			**A**	**B**	**D**	**D**	
**A/Brisbane/59/2007**	**N**	**A**	**S**	**W**	**C**	**S**	**I**	**Y**	**G**	**E**
**A/SalomonIslands/3/2006**	**D**	**A**	**S**	**W**	**C**	**S**	**D**	**Y**	**G**	**E**
A/Corsica/30/2008	D	G	.	.	.	.	D	.	.	Q
A/Corsica/51/2008	D	.	.	.	.	.	D	.	.	.
A/Corsica/75/2008	D	.	N	.	.	.	D	.	.	.
A/Corsica/4/2008	D	G	.	G	.	.	D	F	.	.
A/Corsica/17/2008	D	G	.	G	.	.	D	.	.	.
A/Corsica/29/2008	D	G	.	G	S	C	D		.	.
A/Corsica/34/2008	D	G	N	.	.	.	D	.	.	.
A/Corsica/69/2008	D	.	N	.	.	.	D	.	.	.
A/Corsica/15/2008	D	G	.	.	.	.	D	.	.	Q
A/Corsica/13/2008	D	.	.	.	.	.	D	.	.	.
A/Corsica/12/2008	D	.	N	.	.	.	D	.	.	.
A/Corsica/6/2008	D	G	.	G	S	C	D		.	.
A/Corsica/3/2008	D	G	N	.	.	.	D		.	.
A/Corsica/47/2008	D	.	N	.	.	.	D	.	.	.
A/Corsica/41/2008	D	G	.	.	.	.	D	.	.	Q
A/Corsica/52/2008	D	.	.	.	.	.	D	.	.	.
A/Corsica/76/2008	D	.	N	.	.	.	D	.	.	.
A/Corsica/1/2008	D	G	.	G	.	.	D	F	.	.
A/Corsica/25/2008	D	G	.	.	.	.	D	.	.	Q
A/Corsica/27/2008	D	.	N	.	.	.	D	.	.	.
A/Corsica/13/2008	D	G	.	G	S	C	D		.	.
A/Corsica/26/2008	D	.	.	.	.	.	D	.	.	.
A/Corsica/39/2008	D	G	N	.	.	.	D		.	.
A/Corsica/40/2008	D	.	N	.	.	.	D	.	.	.
A/Corsica/44/2008	D	G	.	.	.	.	D	.	.	Q
A/Corsica/22/2008	D	.	.	.	.	.	D	.	.	.
A/Corsica/11/2008	D	.	N	.	.	.	D	.	.	.
A/Corsica/64/2008	D	G	.	G	.	.	D	F	.	.
A/Corsica/66/2008	D	.	N	.	.	.	D	.	.	.
A/Corsica/72/2008	D	G	.	.	.	.	D	.	.	Q
A/Corsica/80/2008	D	.	.	.	.	.	D	.	.	.
A/Corsica/45/2008	D	G	.	.	.	.	D	.	.	Q
A/Corsica/33/2008	D	.	.	.	.	.	D	.	.	.
A/Corsica/48/2008	D	.	N	.	.	.	D	.	.	.
A/Corsica/7/2007	.	.	.	.	.	.	D	.	R	.

The nucleotide identities between the Corsican A/H1N1 2008 strains and the A/Salomon/3/2006-like lineage were 98.9% and 98.7% based on amino acids. The nucleotide identities between the 2008 strains and the A/Brisbane/59/2007-like lineage were 97.3% and 96.9% based on amino acids.

Seven positions of potential glycosylation with a threshold value of >0.5 were predicted (at positions 15, 27, 58, 91, 129, 164 and 290). These sites were found to be conserved among all isolates in this study.

The nucleotide diversity of this the A/H1N1 2008–2009 population was 0.005±0.002.

### Influenza A/H1N1 pdm virus

**Figure 4:**
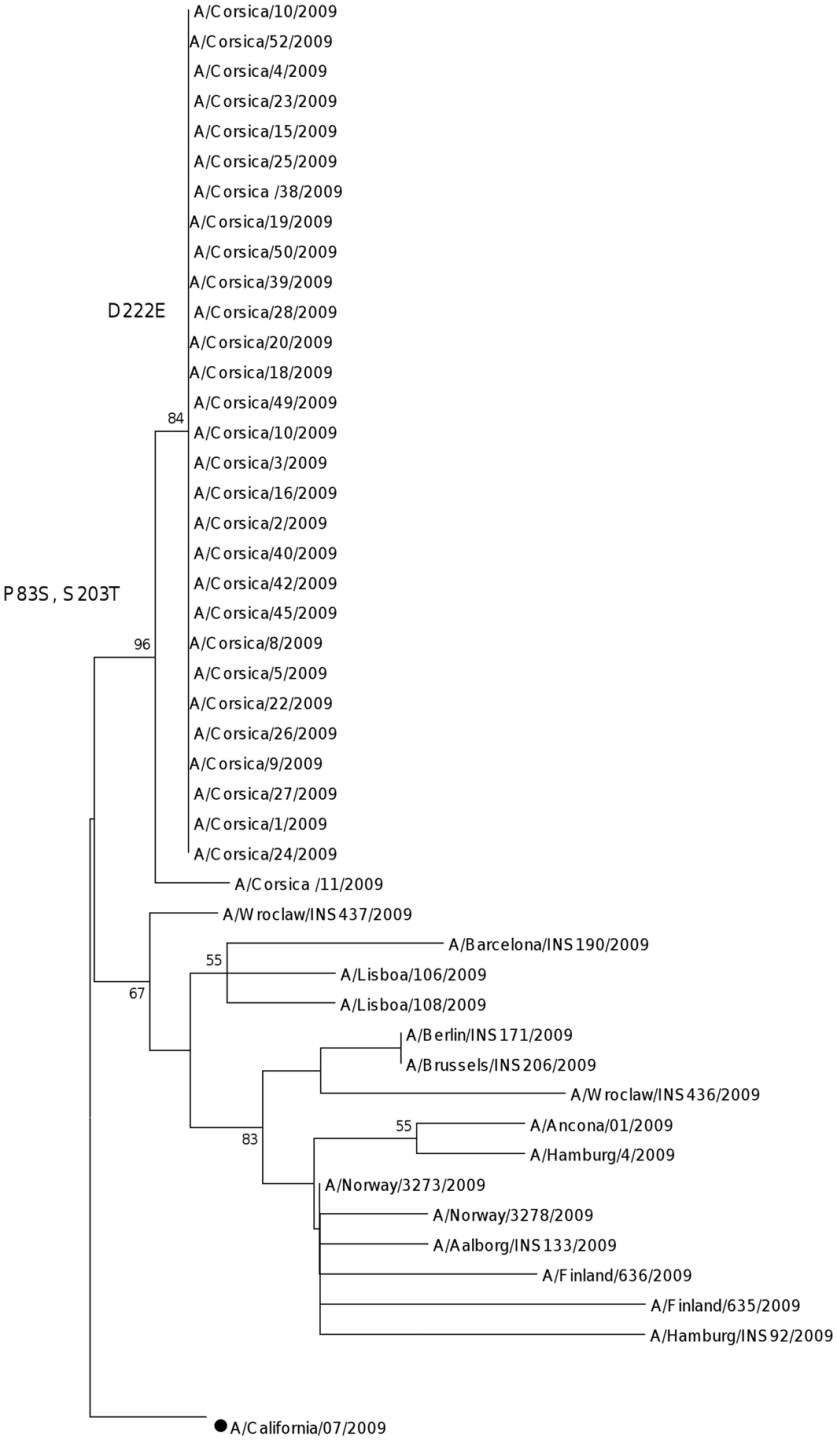
Phylogenetic tree of the HA domain of the A/H1N1 pdm strains from Corsica Island. Maximum likelihood phylogenetic tree analysis of HA genes of A/H1N1 strains circulating in Corsica Island during 2009–2010 season (• represents vaccine strains).

The phylogenetic tree of the HA nucleotide sequences of the Corsican pandemic strains and the vaccine strain is shown in Fig. 4. The H1 phylogeny showed that the amino acid substitutions P83S and S203T were found in all of the Corsican isolates. The substitution D222E was identified in 99% of the A/H1N1pdm viruses (Table 3). The substitution P83S were assigned to epitope E and the substitutions S203T and D222E to epitope D. Eight potential N-glycosylation sites are found in the HA molecule of the A/California/07/2009 virus, six of which reside in the HA1 and the remaining in the HA2 region. Four of six potential glycosylation sites residing in the HA1 region, with a threshold value of >0.5 at positions 28, 40, 104 and 304, were predicted among the A/H1N12009 Influenza virus isolated on Corsica Island.

**Table 3:** Amino acid substitution in A/H1N1 pdm influenza viruses isolated in Corsica Island during 2009–2010.

Amino acid position	32	83	203	222
**Antigenic sites**		E	D	**D**
**A/California/07/2009**	**L**	**P**	**S**	**D**
A/Corsica/01/2009	.	S	T	E
A/Corsica/02/2009	.	S	T	E
A/Corsica/03/2009	.	S	T	E
A/Corsica/04/2009	.	S	T	E
A/Corsica/05/2009	.	S	T	E
A/Corsica/08/2009	.	S	T	E
A/Corsica/09/2009	.	S	T	E
A/Corsica/10/2009	.	S	T	E
A/Corsica/11/2009	I	S	T	
A/Corsica/15/2009	.	S	T	E
A/Corsica/16/2009	.	S	T	E
A/Corsica/18/2009	.	S	T	E
A/Corsica/19/2009	.	S	T	E
A/Corsica/20/2009	.	S	T	E
A/Corsica/22/2009	.	S	T	E
A/Corsica/23/2009	.	S	T	E
A/Corsica/24/2009	.	S	T	E
A/Corsica/25/2009	.	S	T	E
A/Corsica/27/2009	.	S	T	E
A/Corsica/28/2009	.	S	T	E
A/Corsica/36/2009	.	S	T	E
A/Corsica/38/2009	.	S	T	E
A/Corsica/39/2009	.	S	T	E
A/Corsica/40/2009	.	S	T	E
A/Corsica/42/2009	.	S	T	E
A/Corsica/45/2009	.	S	T	E
A/Corsica/48/2009	.	S	T	E
A/Corsica/49/2009	.	S	T	E
A/Corsica/50/2009	.	S	T	E
A/Corsica/52/2009	.	S	T	E

The nucleotide diversity of the A/H1N1pdm isolates in 2009–2010 was 0.002±0.001. The nucleotide identities between the 2009 strains and the A/California/7/2009-like lineage were 99.2% and 99.5% based on amino acids.

### Antigenic distance and vaccine efficacy

**Figure 5:**
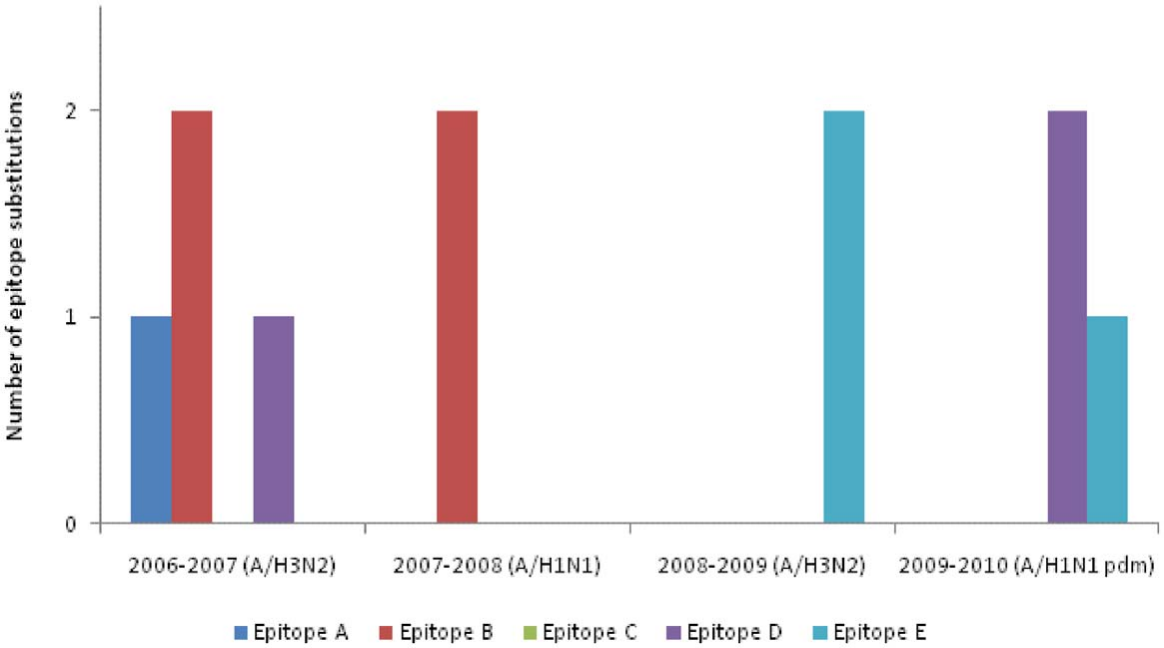
Epitope substitutions over time. Accumulation of epitope substitutions over time in influenza viruses isolated in Corsica Island since 2006.

Vaccine efficacy has a linear correlation with the antigenic distance between the vaccine strain and the circulating virus strains [21]. The accumulation of epitope substitutions over time in influenza viruses strains isolated in Corsica Island are shown in Fig. 5. We calculated the *p*_epitope_ antigenic distance between the 2006–2007 vaccine strain A/Wisconsin/67/2005 and the A/H3N2 Corsican isolates circulating in Corsica Island during 2006–2007 winter season. The largest *p*_epitope_ value was 0.095 (epitope B; substitutions Q156H and L157S), suggesting that two mutations in dominant epitope B would lead to a rate of worst–case VE against these strains of 50.1% (E = 23.5% of 47%, *p*_epitope_ = 0) of that of a perfect match between vaccine and virus. We calculated the antigenic distance from the 2008–2009 vaccine strain A/Brisbane/10/2007 (H3N2) and the A/H3N2 Corsican strains isolated in 2008–2009. Using the *p*_epitope_ method, we found the largest *p*_epitope_ value was 0.045 (dominant epitope = E; substitutions K264P and S265H), suggesting a rate of worst–case VE against these strains of 76% (E = 35.9% of 47%, *p*_epitope_ = 0) of that of a perfect match (*p*_epitope_ = 0) vaccine.

We calculated the antigenic distance from the 2007–2008 vaccine strain A/Salomon Island/3/2006 (H1N1) and A/H1N1 Corsican strains characterized by the mutation S125N (epitope B). Using the *p*_epitope_ method, we found the largest *p*_epitope_ value was 0.045 (dominant epitope = B), suggesting a rate worst–case VE of 90% (E = 47.6% of 53%, *p*_epitope_ = 0) compared of that of a perfect match (*p*_epitope_ = 0) vaccine.

We calculated the antigenic distance from the vaccine strain of the Northern Hemisphere in 2009–2010, A/California/7/2009 and the A/H1N1pdm strains having the substitution P83S, S183T and D222E. The *p*_epitope_ value was of 0.042 (dominant epitope D), suggesting a worst–case VE against these strains of 91% (E = 48% of 53%, *p*_epitope_ = 0) of that of a perfect match between vaccine and virus

## Discussion

Genetic and phylogenetic analysis of influenza A viruses circulating on Corsica Island during the four-year period from 2006–2010 confirmed that the genetic make-up of influenza A viruses may change from year to year.

Our results showed that the strains of influenza A/(H3N2) viruses isolated on Corsica Island belonged to two or more phylogenetic clades co-circulating from the 2006–2007 [25] to 2008–2009 seasons. During the 2006–2007 and 2007–2008 seasons, one clade was dominant. However, in 2006–2007, a sub-clade of A/Nepal/921/06-like circulating strains accumulated variations [25], altering their antigenicity as confirmed by HI assays on representative strains that were conducted by the WHO Influenza Reference Centre (Mill Hill, London). In 2008–2009, isolated viruses had drifted from A/Wisconsin/67/2005-like strains towards the vaccine strain A/Brisbane/10/2007 (vaccine strain for the 2008–2009 season in the Northern Hemisphere). These strains showed a higher value of genetic diversity with respect to the influenza A/H3N2 viruses isolated on Corsica during the 2006–2007 and 2007–2008 seasons.

Variations in the A/H3N2 viruses isolated on Corsica Island were predominantly detected at four antigenic sites A, B, D and E (Table 1). During the 2006–2007 season, a sub-group of isolates was characterized by four mutations previously described in the HA1 sequence of the A/Nepal/921/2006 non-vaccine reference strain and located at antigenic sites A, B and D. Wilson and Cox [26] have proposed that epidemiologically important drift variants usually display four or more amino acid substitutions located at two or more antigenic sites on the HA1 protein. Shih et al. [27] proposed that new antigenic variants are created when more than two mutations occur in antigenic sites or when one variation occurs in one antigenic site and one in a sialic acid RBD. The HA sequences of A/H3N2 viruses isolated on Corsica Island during the 2008–2009 season had drifted from the A/Wisconsin/67/2005-like lineage. These strains, which were characterized by two fixed mutations located at site E (K264E and K265E) compared to the vaccine strain A/Brisbane/10/2007, were closely related to the A/Brisbane/10/2007-like lineage (vaccine strain for the 2008–2009 season in the Northern Hemisphere). Recently, Huang et al. [28] showed that a pair of viruses are often “similar viruses” if the epitope A and B, which are closed to the RBD, are not changed.

Few mutations in the A/H1N1 seasonal isolates during the 2007–2008 season that differentiate them from the A/SalomonIslands/3/2006 (S125N at antigenic site B) and A/Brisbane/59/2007-like (S189D fixed at antigenic site B) lineages were located at the antigenic sites (Table 2).

These findings seem to indicate that non-synonymous changes in the HA1 gene, especially in antigenic regions, have been more frequent in A/H3N2 than in seasonal A/H1N1 in influenza virus isolated in Corsica Island.

Effectively, since the reintroduction of seasonal A/H1N1 into human populations in 1977, this subtype has exhibited a lower case fatality rate than A/H3N2, particularly when these subtypes co-circulate [29]. In addition, A/H1N1 undergoes less severe seasonal genetic bottlenecks than A/H3N2, resulting in less pronounced or frequent reductions in genetic diversity [30].

This hypothesis was supported by subtype-specific differences in adaptation rate with higher rates of adaptation for A/H3N2 than A/H1N1 in HA and NA genes [31].

The A/H1N1pdm isolates were characterized by one fixed mutation at site E and two fixed mutations at site D with respect to A/California/07/2009 (Table 3). All Corsican A/H1N1pdm strains analyzed were characterized by the S203T mutation specific to clade 7 isolates [32]. The clade 7 marker variation S203T, observed in all sampled Corsican viruses, is a A/H1N1pdm virus site under positive selection and also involved in antigenicity [33].

The viruses constituting this clade were therefore responsible for most of the pandemic burden worldwide. Following its origin, which remains obscure, the clade 7 viruses have been subjected to strong purifying selection, with the exception of the earliest phases of its evolution, behaving later as a well-fit virus, similar to viruses circulating in swine or seasonal influenza in humans.

Overrepresentation of certain mutations among geographically and temporally related samples needs to be carefully controlled for possible founder effects which could be identified as homogenous clusters in phylogenetic analyses, as was observed to be the case for the D222E mutation. Phylogenetic analysis of A/H1N1pdm influenza viruses showed that viruses isolated in Corsica Island formed a separate sub-clade of clade 7 as a consequence of the presence of the D222E substitution. While founder effect mutations cannot automatically be linked to phenotypes simply by increased occurrence, they may nevertheless alter the virus fitness for which even tiny changes could result in advantages shifting selection to their favor [34]. Recent studies showed that this position was positively selected for human strains [35]–[36]. Moreover, this codon, located in antigenic site D, is also associated with RBD indicating a positive selection from the hosts, caused maybe by vaccination and mass use of antiviral drugs.

This mutation, whose biological meaning is still unknown, has been isolated in other countries but at lower frequencies (e.g.; Italy, Turkey, Sweden and Finland). Recently, a cluster of D222E viruses among school children was isolated in Italy, confirming human-to-human transmission of viruses mutated at amino acid position 222 [37].

The D222G mutation was not observed among A/H1N1pdm isolated on Corsica Island. This substitution was observed more frequently in viruses isolated from patients with fatal outcomes [38].

Several studies showed that the A/H1N1pdm virus has a high genome-wide evolutionary rate (3.6661023 substitutions/site/year) [39] and a rate for the HA segment (0.961023 substitutions/site/year) that represents the lowest of all of the viral HAs of seasonal A/H1N1 (1918–1957; 2.961023), seasonal A/H1N1 (1977–2009; 1.761023), and swine H1 (1930–2009; 1.961023) [33].

All Corsican A/H1N1pdm isolates possessed D204 in the RBD, which confers binding of H1 viruses to human receptors, supporting efficient transmission of these viruses in humans. Key residues in the RBD [40] predicted to have a role in binding to human receptors (T98Y, S136T, 153W and 183H) were found to be Y98, S136, W153 and H183 in the Corsican isolates as in other A/H1N1pdm viruses isolated elsewhere.

Among the A/H3N2 Corsican isolates, the residues mainly for NeuAcα2,6Gal linkage specific for the H3 subtype were Ile(I)226 and Ser (S)228, similar to other studies [41].

N-linked glycosylation is conserved among various HA subtypes of influenza A viruses. Its presence or absence can cause an increase or loss in function of the glycoprotein because N-linked glycosylation can initiate and maintain folding, stability, solubility, antigenicity and immunogenicity of the protein. Most of the currently circulating viruses have six or seven N- glycosylation sites in the HA globular head region. Interestingly, the predicted N-linked glycosylation site at position 144 of the HA antigenic site A has been observed in the majority of strains isolated on Corsica in 2006–2007 and was definitively lost during the 2007–2008 and 2008–2009 seasons. Other studies have reported the loss of this predicted N-linked glycosylation site in all strains isolated since 2006–2007 [42].

In this study, we tried to predict a rate of VE for A/H3N2 and A/H1N1 seasonal influenza viruses and of the A/H1N1pdm virus using the *p*_epitope_ model. This model has been developed to provide researchers and health authorities with a new tool to quantify antigenic distance and to help with vaccine design. These results showed that the rate of VE compared to that of a perfect match against influenza viruses circulating on Corsica Island varied substantially across the four seasons analyzed, and that tend to be highest for A/H1N1 compared with A/H3N2 vaccines, suggesting that cross-immunity seems to be stronger for H1 HA gene [23].

Even if, as precedently described [21], the vaccine effectiveness estimated by several epidemiologic studies seems to be supported by the *p*_epitope_ model, the rate of VE estimated in this study need to be boosted with real VE data from the same patients. Nevertheless, studying the relationship between epitopes and vaccine efficiency [21] could be useful for studying influenza virus evolution and to consolidate studies based on epidemiological data.

One important limit of this study is that we have not analyzed the genetic evolution of the influenza virus outside HA proteins. Suzuki (2008) [43] applied a dN/dS approach to 100 complete H3N2 genomes and concluded that negative selection dominated in all proteins; significant positive selection was observed only in a handful of codons in the HA, NA and NP genes. Pond et al., (2008) [44] analyzed the same data using a technique more sensitive to the detection of individual selective sweeps and reported more evidence for adaptation in 5 genes: PB2 (2 codons), PB1 (5 codons), PA (3 codons), HA (81 codons), and NA (4 codons).

A recent study [31] showed that the rate of adaptation (per codon per year) is higher in surface residues of the viral NA than in HA1, indicating strong antibody-mediated selection on the former. They also observed high rates of adaptive evolution in several non-structural proteins, which may relate to viral evasion of T-cells and innate immune responses [31].

Another limit of the study is based on the sensitivity and specificity of molecular techniques used to identify strains and on the quality of specimens analyzed. Even if, the M RT-PCR used in this study to identify influenza A virus is specific and sensitive, there is a limit of detection between 10 and 100 copies/reaction [15].

The molecular analysis of the HA gene of influenza viruses that circulated on Corsica Island between 2006–2010 showed for each season the presence of a dominant lineage characterized by at least one fixed mutation. It must be noted that A/H3N2 and A/H1N1pdm were characterized by multiples fixation at antigenic sites. Subsequent mutations at antigenic sites often significantly contribute additional effects, so that multiple fixations, possibly including hitchhikers, occur rapidly and (almost) simultaneously. The fixation of specific mutations at each outbreak could be explained by the combination of compensatory mutations, neutral phenomenon and a founder effect, favoring the presence of a dominant lineage in a closed environment such as Corsica Island.

These findings confirmed that careful surveillance of genetic changes in the HA1 domain during the influenza epidemic season may provide early information on virus variants and improve the influenza vaccine.
